# Catastrophic costs of tuberculosis care in a population with internal migrants in China

**DOI:** 10.1186/s12913-020-05686-5

**Published:** 2020-09-04

**Authors:** Liping Lu, Qi Jiang, Jianjun Hong, Xiaoping Jin, Qian Gao, Heejung Bang, Kathryn DeRiemer, Chongguang Yang

**Affiliations:** 1 Department of Tuberculosis Control, Songjiang District Center for Disease Control and Prevention, Shanghai, China; 2 Key Laboratory of Medical Molecular Virology (MOE/NHC/CAMS), School of Basic Medical Sciences, Shanghai Medical College, Fudan University, Shanghai, China; 3 Department of Public Health Sciences, University of California, Davis, CA USA; 4 Department of Epidemiology of Microbial Diseases, Yale School of Public Health, Yale University, 60 College Street, New Haven, CT 06510 USA

**Keywords:** Tuberculosis, Migrant, Catastrophic cost, Economic burden, China

## Abstract

**Background:**

The internal rural-to-urban migration is one of the major challenges for tuberculosis (TB) control in China. Patient costs incurred during TB diagnosis and treatment could cause access and adherence barriers, particularly among migrants. Here, we estimated the prevalence of catastrophic costs of TB patients and its associated factors in an urban population with internal migrants in China.

**Methods:**

A cross-sectional survey was conducted to enroll culture-confirmed pulmonary TB patients in Songjiang district, Shanghai, between December 1, 2014, and December 31, 2015. Consenting participants completed a questionnaire, which collected direct and indirect costs before and after the diagnosis of TB. The catastrophic cost was defined as the annual expenses of TB care that exceeds 20% of total household disposable income. We used logistic regression to identify factors associated with catastrophic costs.

**Results:**

Overall, 248 drug-susceptible TB patients were enrolled, 70% (174/248) of them were from migrants. Migrant patients were significantly younger compared to resident patients. The total costs were 25,824 ($3689) and 13,816 ($1974) Chinese Yuan (RMB) in average for resident and migrant patients, respectively. The direct medical cost comprised about 70% of the total costs among both migrant and resident patients. Overall, 55% (132 of 248) of patients experienced high expenses (>10% of total household income), and 22% (55 of 248) experienced defined catastrophic costs. The reimbursement for TB care only reduced the prevalence of catastrophic costs to 20% (49 of 248). Meanwhile, 52% (90 of 174) of the internal migrants had no available local health insurance. Hospitalizations, no available insurance, and older age (> 45-year-old) contributed significantly to the occurrence of catastrophic costs.

**Conclusions:**

The catastrophic cost of TB service cannot be overlooked, despite the free policy. Migrants have difficulties benefiting from health insurance in urban cities. Interventions, including expanded medical financial assistance, are needed to secure universal TB care.

## Introduction

Tuberculosis (TB) is often recognized as a disease of poverty and is known to disproportionately affect the economically disadvantaged population [1]. One of the three goals of End TB strategies by 2030 includes reducing the catastrophic costs to TB-affected families [1, 2]. This goal is a challenge as more than 50% of TB patients have experienced financial difficulties due to the direct and indirect costs caused by TB care [3], particularly in countries with a high-TB burden such as China and India. China has the second-largest TB burden worldwide, with about one million incident TB cases annually [4]. Currently, the increase in internal rural-to-urban migrants is one of the major challenges for TB control in China. Most of the internal migrants are men who leave the rural countryside to join the wage economy in towns and cities all over China. There are an estimated 240 to 260 million internal migrants in urban areas of China by 2030 [5]. However, the household registration (*Hukou)* system legally ties the internal migrants and their family members to their rural homes and bars them from receiving most public benefits in cities. As a result, internal migrants are not entitled to subsidized housing or education, have poor access to social security, health insurance, and medical benefits, often share crowded living conditions due to low socio-economic status, and are thus more vulnerable to communicable disease including TB [6–9].

Patient costs incurred during TB diagnosis and treatment, as well as seeking and receiving health care, could cause barriers to access and adherence that can affect treatment outcomes and increase the risk of transmission of disease [10]. This could hamper TB control, particularly for vulnerable populations such as immigrants (internal and international migrants). In China, people with TB-related symptoms can have free sputum smear tests and a primary treatment package if TB is confirmed. However, there are many out-of-pocket health care costs associated with TB diagnosis, hospital admissions, and treatment, which were associated with high financial burden and low adherence [10–14]. In the recent past, internal migrants could not share the free TB policy in urban centers, and they mostly were uninsured of social health insurance or not allowed to use their health insurance scheme (e.g., new rural cooperative medical scheme) in urban cities [15], only in the town or village where they were born. Although some urban cities like Shanghai have expanded the health care services to offer free TB diagnosis and treatment to include internal migrants in urban areas since 2004, there are still medical charges for TB diagnosis and treatment (e.g., liver protection medicine) [13]. Meanwhile, out-of-pocket payments for transportation, accommodation, and food to get access to TB diagnosis and treatment could also have a significant impact on the household economic burden of TB patients [16]. Here, we hypothesized that internal migrants incurred a higher economic burden during the TB care in urban centers, partially due to the barriers to accessing the health system and insurance benefits. We conducted a cross-sectional study to estimate the occurrence of catastrophic cost of TB care and to identify its associated factors among both internal migrants and residents in an urban area in China.

## Methods

### Study site and population

We designed and conducted a cross-sectional survey on the economic burden for TB care in Songjiang, Shanghai, from December 1, 2014, to December 31, 2015. Songjiang is one of 15 districts in Shanghai and had a rapid increase in the proportion of internal migrants among its population since the nationwide economic reforms. In 2013, Songjiang had a GDP of US$15.04 billion and ranked first in the export volume in Shanghai. In 2015, 1.1 million (60%) of the total population in the Songjiang district were internal migrants [17]. Shanghai has a well-established TB service delivery system that consists of a three-level (consists of CDC, designated TB hospital, and community health services center) referral network [7]. Community physicians routinely screened and referred individual with TB-like symptoms to the local designated TB hospital for diagnosis. This study continuously enrolled bacteriologically confirmed pulmonary TB patients among both residents who had Shanghai *Hukou* and internal migrants who had *Hukou* of other Chinese provinces and had lived in Shanghai for at least six months. Prisoners and mentally ill/cognitively impaired and institutionalized TB patients were excluded from this study due to unavailable cost estimates and ethical concerns. Eligible patients who completed their treatment during the study period were invited to a face-to-face interview by trained community health workers after they signed the informed consent forms.

### Diagnosis and treatment of TB

Beginning in 2004, the Shanghai Centers for Disease Control and Prevention (CDC) implemented a new policy extending free TB treatment to all migrants. During the period of our study, all individuals with TB-like symptoms had three sputum samples collected (spot, early morning, and night) and tested for the presence of *Mycobacterium tuberculosis* (*M. tuberculosis*) by microscopic exam and culture. We used sputum induction for individuals who were unable to produce sputum spontaneously. From each individual with culture-positive TB, a single pre-treatment sputum specimen was submitted to the Shanghai Municipal CDC TB reference laboratory for rifampin (RIF) and isoniazid (INH) drug susceptibility testing (DST) by the proportion method on Löwenstein-Jensen medium. Multidrug-resistant TB (MDR-TB) patients were excluded in this study, as the costs for MDR-TB were much higher than drug-susceptible TB, and the rate of MDR-TB in Songjiang is relatively low [18]. In the study district, once being confirmed as pulmonary TB, patients were asked to sign an agreement with the local CDC that they could get reimbursement for first-line anti-TB drugs and certain times of TB related tests using their medical receipts once they complete the treatment period.

### Data collection

We collected data on TB patients’ direct and indirect costs from their activities to seek TB diagnosis and during TB treatment using a standardized questionnaire, modified from the Poverty sub-working group of the Stop-TB Partnership, titled “Tool to Estimate TB Patient’s Costs” [19]. We conduct a pilot interview with ten enrolled patients to adjust and change the expression of the questions in much more understandable and local fit language. This survey also collected additional demographic information and routine data on diagnostic delays, complications, sputum smear test results, and the type of TB diagnosed. Patients’ medical receipts were used as source documents to calculate the sum of different kinds of medical costs, including registration fees, test costs, drug costs, et al. (Supplementary). Those who lost medical receipts for over two times during TB care-seeking were excluded from the study.

### Measurements and definitions

Direct costs were the out-of-pocket payments for TB care (medical costs) and costs incurred in the pathway to care, to access the services, including transportation, food, and accommodation costs. Indirect costs accrued from patients’ lost income, which was calculated by multiplying the local standard monthly (2020 RMB monthly in 2014) income to the total lost time for an individual to receive a diagnosis, treatment, and care. Guardian costs were the costs incurred by family members looking after or caring for the patient during the patients’ treatment. The total TB costs were the sum of a patient’s direct, indirect, and guardian costs. The out-of-pocket (OOP) costs were calculated by subtracting insurance payments from the total TB costs. The affordability of costs was measured as the proportion of OOP cost compared to the patients’ annual household disposable income. The costs were reported in Chinese Yuan (RMB), as well as US Dollars (USD) under the exchange rate for USD/RMB in 2015 of 6.2837. We defined catastrophic expenditure if the patient’s OOP cost exceeds 20% of the annual household income according to the WHO recommendation [20]. A diagnostic delay was defined as the time interval (days) from the onset of symptoms to the date of diagnosis. A treatment delay was defined as the time interval (days) from the onset of symptoms to the initiation of treatment. According to the Chinese TB control guidelines, a bacteriologically confirmed TB patient would be regarded as “cured” if his/her sputa are tested negative by smear for sequential twice times at the end of the treatment, while would be defined as “completed treatment” if he/she does not have enough sputum test results.

### Statistical analysis

We summarized the demographic characteristics and costs for all participants. TB related costs were described by the mean and interquartile range (IQR). We compared groups using the chi-square test for categorical variables (including a linear trend test of the odds) and the Wilcoxon nonparametric rank-sum test for continuous variables. We used multivariates logistic regression analyses to evaluate potential risk factors associated with catastrophic cost among internal migrants and residents. In addition, a sensitivity analysis was performed by comparing a 10% of annual income threshold with household status and other covariants, as suggested in the WHO handbook for cost survey [20] All *p*-values were two-sided, and statistical analyses were performed using the Stata/SE version 13.1 (StataCorp, College Station, TX, USA).

## Results

### Study population

From December 1, 2014, to December 31, 2015, we continually enrolled 252 culture-confirmed pulmonary TB patients diagnosed in the Songjiang district, Shanghai. Of the 252 cases enrolled, four (1.6%) were excluded: three patients had an infection with non-tuberculosis mycobacteria (NTM), and one other case died of respiratory failure during the anti-TB treatment. Among the remaining 248 participants whose data were included for analyses, 29.8% (74 of 248) were residents, and 70.2% (174 of 248) were internal migrants. The characteristics of the study population were detailed in Table 1.

**Table 1:** Demographic, socio-economic and clinical characteristics of Tuberculosis patients among residents and migrants

Variable	Total		Residents		Migrants		*p* value
	*N* = 248		*N* = 74		*N* = 174		
Age, yrs. [median (IQR)]	34	(26–49)	52	(34–62)	32	(25–40)	< 0.01
Gender [n (%)]							0.40
Male	167	(67.3)	47	(63.5)	120	(69.0)	
Female	81	(32.7)	27	(36.5)	54	(31.0)	
Education level [n (%)]							0.01
Primary	46	(18.5)	19	(25.7)	27	(15.5)	
Middle	88	(35.5)	20	(27.0)	68	(39.1)	
High	82	(33.1)	20	(27.0)	62	(35.6)	
College	32	(12.9)	15	(20.3)	17	(9.8)	
Living area per capita, m^2^ [median (IQR)]	16	(9–30)	34	(24–60)	12	(8–20)	< 0.01
No. of member per household [median (IQR)]	3	(3–5)	3	(3–4)	4	(3–5)	0.15
Mean Income, thousand RMB [median (IQR)]							
Monthly income	3.5	(2.0–4.5)	2.9	(1.3–3.0)	3.8	(2.5–4.9)	< 0.01
Annual household income	80	(54–120)	70	(50–100)	90	(60–120)	0.01
Annual household income per capita	24	(16–33)	20	(15–32)	25	(17–36)	0.06
Medical insurance status [n (%)]							< 0.01
Yes	154	(62.1)	70	(94.6)	84	(48.3)	
No-feasible (self-pay)	94	(37.9)	4	(5.4)	90	(51.7)	
Debts [n (%)]							0.96
Yes	17	(6.9)	5	(6.8)	12	(6.9)	
No	231	(93.1)	69	(93.2)	162	(93.1)	
Occurence of Catastrophic cost							< 0.01
Yes	55	(22.2)	25	(33.8)	30	(17.2)	
No	193	(77.8)	49	(66.2)	144	(82.8)	
TB history [n (%)]							0.03
Yes	17	(6.9)	9	(12.2)	8	(4.6)	
No	231	(93.1)	65	(87.8)	166	(95.4)	
Number of patient visits [median (IQR)]							
Pre-diagnosis	3	(2–4)	3	(2–4)	3	(2–4)	0.17
Post-diagnosis	14	(11–17)	15	(12–19)	13	(11–16)	0.01
Treatment outcomes [n (%)]							0.80
Cured	85	(34.3)	26	(35.1)	59	(33.9)	
Completed	158	(63.7)	46	(62.2)	112	(64.4)	
Others	5	(2.0)	2	(2.7)	3	(1.7)	
Sputum smear microscope testing [n (%)]							0.82
Positive	88	(35.5)	27	(36.5)	61	(35.1)	
Negative	157	(63.3)	45	(60.8)	112	(64.4)	
Default	3	(1.2)	2	(2.7)	1	(0.6)	
Hospitalization [n (%)]							< 0.01
None	163	(65.7)	31	(41.9)	132	(75.9)	
Yes	85	(34.3)	43	(58.1)	42	(24.1)	
Pre-diagnosis	56	(22.6)	26	(35.1)	30	(17.2)	
Post-diagnosis	18	(7.3)	11	(14.9)	7	(4.0)	
In both period	11	(4.4)	6	(8.1)	5	(2.9)	
Diagnosis delay, days [median (IQR)]							
Patient delay (days from symptom to seeking care)	10	(3–22)	10	(3–26)	10	(2–20)	0.17
Hospital delay (days from seeking care to diagnosis)	13	(6–20)	15	(7–22)	11	(6–19)	0.07

* IQR = Interquartile range; RMB, Chinese Yuan

Internal migrant TB patients were significantly younger than resident cases (median age, 32 years *vs.* 52 years; *p* < 0.01), had a higher annual household income (median 90,000 *vs.* 70,000 Chinese Yuan [14,323 *vs.* 11,140 US Dollars], *p* = 0.01). However, migrants with TB had a significantly smaller living space per capita compared to residents (12 [IQR, 8–20] *vs.* 34 [IQR, 24–60] square meters, *p* < 0.01). Half (52%, 90 of 174) of the migrant cases had no health insurance and paid all of the medical costs out-of-pocket by themselves. In contrast, most (95%, 70 of 74) residents had health system insurance that covered the related medical expenditures. Also, internal migrants had a significantly lower rate of hospitalizations than that of resident patients during both the periods before and after the diagnosis of TB (24%, 42 of 174 *vs.* 58%, 43 of 74, *p* < 0.01).

Most of the TB patients were either cured (34%, 85 of 248) or completed the treatment (64%, 158 of 248) during the study period, and such proportions were similar between internal migrant and resident TB patients. Patients went to the hospital for an average of three times before being diagnosed with TB and went to the hospital 14 more times during TB treatment. The patient delayed an average of 10 [IQR 3–22] days to seek medical care after well-defined TB symptoms occurred, and the hospital delayed an additional 13 [IQR 6–20] days to make the diagnosis.

### Direct costs and indirect costs of TB services

**Fig. 1:**
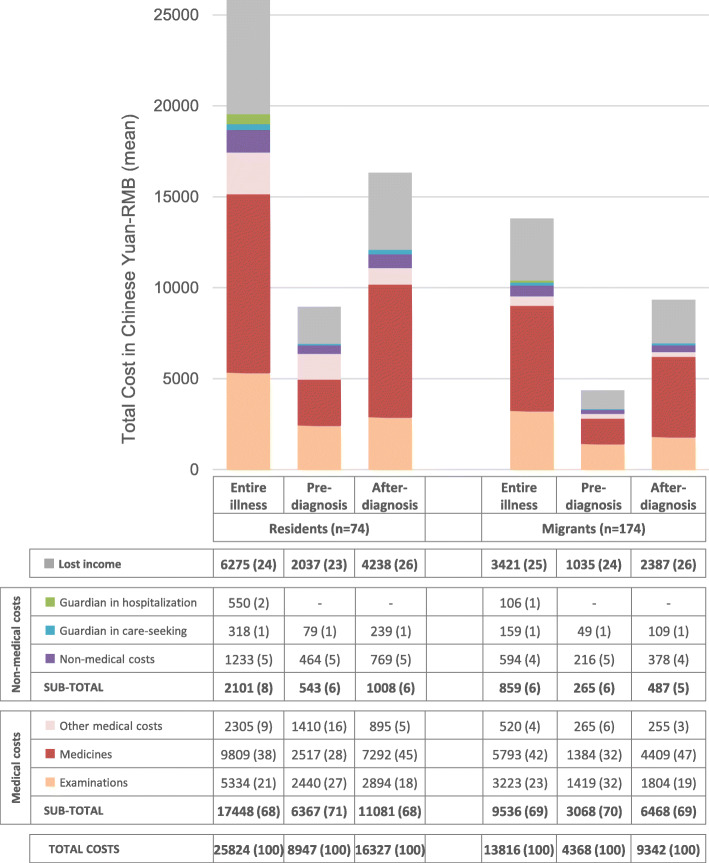
Patient cost by treatment stage in mean Chinese Yuan (RMB), stratified by migrant status. The bar color corresponds to the color of cost categories in the table

The direct costs and indirect costs (i.e., lost income) are summarized in Fig. 1. In general, residents paid almost twice as much as internal migrants for direct costs and lost income (25,824 and 13,816 Chinese Yuan [4,110 *vs.* 2,199 US Dollars], respectively). The majority of the total expenses (> 65%) were direct medical costs. The proportion of total costs that were direct medical costs was similar among residents and internal migrants (67.6 *vs.* 69.0%). Non-medical items, including transportation, extra food supplies, accommodation, and guardian care, accounted for a higher proportion of total costs among residents compared to internal migrants (8.1% [95%CI 7.8–8.5] *vs.* 6.2% [95%CI 5.8–6.6], *p* < 0.01). The other quarter of total costs to TB patients were from indirect income loss. The proportion of indirect costs among the total costs did not differ significantly between residents and internal migrants (24.3 *vs.* 24.8%, *p* = 0.30).

### Costs incurred among different treatment stages

The costs accrued before getting a TB diagnosis were one-third of the total expenses and its proportion was slightly higher among residents compared to internal migrants (35.4% [95%CI 34.8–36.0] *vs.* 31.9% [95%CI, 31.1–32.6], *p* < 0.01). The proportion of lost income increased, and medical costs decreased slightly after diagnosis; meanwhile, the costs of medicine after diagnosis rose from 28.1 to 44.7% of the total expenses among residents and from 31.7 to 47.2% among internal migrants.

### Catastrophic costs and risk factors

Overall, 55 (22%, 55 of 248) TB patients have experienced household catastrophic costs during the entire illness (Table 1). The proportion of catastrophic costs was significantly different between households of residents versus households of internal migrants (*p* < 0.01, Table 1). However, after adjustment for hospitalization, the health insurance, and age groups, there was no significant difference in the prevalence of catastrophic costs between migrants and residents (adjusted OR [aOR], 0.64, 95%CI [0.25–1.61], Table 2). In the multivariate regression model, TB patients who experienced hospitalization (aOR, 10.08, 95%CI [4.96–23.41]), having no available health insurance (aOR, 2.69, 95%CI [1.68–6.72]), and being older than 45 years (aOR, 2.50, 95%CI [1.20–5.21]) were independently associated with the occurrence of catastrophic costs (Table 2).

**Table 2:** Risk Factors associated with catastrophic cost before (CC1) and after (CC2) the TB specific reimbursement

	Univariable analysis, CC1		Multivariable logistic regression, CC1		Multivariable logistic regression, CC2	
	OR (95%CI)	p value	Adjusted OR (95%CI)	p value	Adjusted OR (95%CI)	p value
Migrants	0.41 (0.21–0.77)	< 0.01	0.64 (0.25–1.61)	0.34	0.81 (0.31–2.13)	0.68
Sex, females	1.00 (0.53–1.90)	0.99				
Age, years	1.04 (1.02–1.06)	< 0.01				
More than 45 years old	3.87 (2.02–7.43)	< 0.01	2.50 (1.20–5.21)	0.01	2.52 (1.17–5.47)	0.01
Primary education and lower	3.24(1.60–6.58)	< 0.01				
Living area per capital, m^2^	1.01 (1.00–1.02)	0.01				
Family size (per person increased)	0.84 (0.65–1.10)	0.19				
Unemployment	2.17 (0.86–5.49)	0.10				
No feasible health insurance	1.12 (0.60–2.07)	0.36	2.69 (1.08–6.72)	0.03	2.33 (0.89–6.14)	0.08
Previous TB episode	2.67 (0.96–7.45)	0.06				
Patient delay; days	1.01 (0.99–1.02)	0.07				
Hospital delay; days	1.00 (0.99–1.01)	0.76				
Smear positive	1.17 (0.62–2.21)	0.63				
Hospitalization	11.27 (5.55–22.89)	< 0.01	10.78 (4.96–23.41)	< 0.01	14.92 (6.30–35.28)	< 0.01

Note, CC1 represents catastrophic costs without reimbursement from local CDC, and CC2 represents catastrophic expenditures after reimbursement from local CDC; OR = odds ratio

The average proportion of the total costs that were covered by insurance payments was 18.8% (95%CI [14.0–23.7]) among internal migrants compared to 54.0% (95%CI [48.5–59.4]) of residents. The benefits of reimbursement for TB diagnosis and treatment had less impact on the prevalence of the catastrophic cost by reducing it from 22 to 20% (49 of 248, *p* = 0.50). Besides, TB patients who experienced hospitalization and were older than 45 years remained significant for catastrophic costs after this specific reimbursements, except the health insurance (Table 2).

We performed sensitivity analyses by using a threshold of total costs of 10% or more of annual income. The proportion of patients incurred catastrophic costs increased sharply to 46% (116 of 248). No significant difference in this proportion was observed among migrants and residents. When this threshold was used in the multivariable regression model, it was found that the 10% defined catastrophic cost was not independently associated with health insurance. Of note was that the increase of family size per person had protection in incurring catastrophic cost (aOR,0.72, 95%CI[0.57–0.90]) after adjusting for migration status, hospitalization, and age more than 45-year-old.

### Hospitalization and health insurance among migrants and residents

Hospitalization rate was significantly higher among resident patients (58%, 43 of 74) compared to migrant patients (24%, 42 of 174, *p* < 0.01, Table 3). Migrant patients had a significant trend association between hospitalization and age (p < 0.01, Table 3), while resident TB patients had a high average rate without the trend association (*p* = 0.59, Table 3). Overall, patients without health insurance were less likely to be hospitalized than those with health insurance (24%, 23 of 94 *vs.* 40%, 62 of 154, *p* = 0.01). In a multivariable logistic regression model, TB patients who were a migrant (aORs, 0.29, 95%CI [0.14–0.56]) and age between 25 and 34 years old (aORs, 0.28, 95%CI [0.12–0.67]) were less likely to experience hospitalization. Since only 5% of the resident patients lacked health insurance, we stratified this analysis by migration status; however, we did not observe any significant factors for the prevalence of hospitalization in either resident or migrant patients subgroups.

**Table 3:** Distribution of TB patients with hospitalization by age groups, migrant strata, and health insurance status

Age groups	TB Patients with hospitalization									
	Total (%)		Migrant TB patient (%)		Resident TB patient (%)		With insurance (%)		Self-pay* (%)	
Total	85/248	(34)	42/174	(24)	43/74	(58)	62/154	(40)	23/94	(24)
15 ~ 24	18/46	(39)	13/39	(33)	5/7	(71)	10/20	(50)	8/26	(30)
25 ~ 34	14/81	(17)	6/65	(9)	8/16	(50)	12/54	(22)	2/27	(7)
35 ~ 44	10/38	(26)	6/31	(19)	4/7	(57)	8/25	(32)	2/13	(15)
45 ~ 54	13/34	(38)	8/22	(36)	5/12	(42)	8/17	(47)	5/17	(29)
55 ~ 64	18/31	(58)	6/12	(50)	12/19	(63)	13/22	(59)	5/9	(56)
65+	12/18	(67)	3/5	(60)	9/13	(69)	11/16	(69)	1/2	(50)

* Most (90 of 94) of the self-pay TB patients were from migrant

## Discussion

Despite the free TB care policy, TB patients among both internal migrants and residents incurred high costs due to TB care and a high rate of catastrophic costs in the Songjiang district, Shanghai, China. Internal migrants, who were mainly from rural areas, accounted for the majority of the urban TB disease burden. They were less likely to benefit from health insurance during their medical care in urban cities and were less likely to be hospitalized, and had a comparable risk to experience household catastrophic cost compared to resident patients.

We defined total catastrophic costs due to TB as the sum of total costs to patients that exceeded 20% of the annual household income. However, there is no universal standard for this definition, and a range of thresholds (5–25%) of total household incomes was used in other studies [14, 21, 22]. A prospective study in Peru analyzed the relationship between prognosis and treatment costs in patients with MDR-TB and defined clinically relevant catastrophic costs with a 20% threshold [23]. Studies from China used more than 10% of annual income or more than 40% of non-food income or capacity to pay as the cutoff to define the catastrophic costs and reported a high rate from 60 to 90% in the rural regions [14]. In current study, the rate of catastrophic costs was 22% with a 20% threshold but increased sharply to 46% if a 10%-threshold being applied. Catastrophic costs can lead to reduced access to health care and completion of TB treatment, as well as poverty reinstatement due to illness. We showed that more than one-fifth of the TB patients in the Songjiang district in Shanghai had catastrophic expenditures, regardless of their household status (i.e., being an internal migrant or a resident). This finding indicates that the proportion of catastrophic expenditures due to TB disease should not be ignored, and the current health and social security system needs more efforts toward the goal of zero catastrophic expenditure for TB disease in 2030 [1].

Of note, the internal migrant TB patients had a slightly higher average income compared to that among residents. It’s mainly because the majority of the migrant patients were young individual workers (e.g., service workers and labor workers). In contrast, most of the resident patients were older adults who lacked decently paid work or were retired. Despite their relatively high income, there was no significant difference in the risk of the catastrophic costs between migrant and resident TB patients after adjusting for age, hospitalization, and household income. It has been widely reported that internal migrants faced many barriers to seeking TB care. Although we did not observe significant differences in the delays in diagnosis and treatment outcomes between internal migrants and residents, we cannot ignore the high economic burden caused by TB care.

Another notable finding is that more than half of the migrants with TB had to pay their medical costs by themselves instead of paying through urban health insurance, mainly because of the household registration system in China. To reduce their medical costs for TB, Shanghai and many other cities with large increasing populations of internal migrants (e.g., Beijing, Shenzhen, and Hangzhou) expanded the free TB treatment policy to migrants around 2004–2006. In the Songjiang district, TB patients who finished the treatment period could get the reimbursement that was financially supported by the local central government. However, such reimbursement, which only covered TB-related testing during the first medical visit and the first-line anti-TB drugs, had a limited effect on reducing the rate of catastrophic costs for both migrants and residents. Besides, the reimbursement system requires migrants to provide a residential permit to qualify for this reimbursement, and the permit became mandatory beginning in 2018.

Hospitalization during the TB treatment accounted for more than 60% of the health care expenditures. Residents were about four times more likely to be hospitalized than migrants. Migrants tend to avoid hospitalization, regardless of whether they can use their health insurance or not in an urban city. The relatively lower frequency of hospitalization among migrant TB patients could also be partially due to the much younger population of migrants. It is also reasonable to infer that the type of health insurance that migrants had could impact their healthcare-seeking behavior [24].

The free TB policy in China only covers the first time diagnosis and first-line anti-tuberculosis drugs. The effectiveness of the implementation also variates in different areas, and many costs are still not covered [16]. Further, in Shanghai, all expenses need to be paid in advance, and some of the costs can only be reimbursed after the completion of anti-TB treatment. From the patient management perspective, this necessary arrangement offers a financial incentive for TB patients to complete therapy and to improve the provider’s treatment completion rate. But various types of certificates are required for internal migrants to obtain the TB reimbursement, including the residence permit, which also creates barriers. In contrast, some implementation studies refer to the use of transportation subsidies (50–100 RMB or 7–14 USD) and monthly living allowances (100–200 RMB or 14–28 USD) for migrant patients to increase treatment compliance [25]. However, the amount of subsidies is relatively low for the overall medical expenses, and it is difficult to alleviate the medical burden of patients. In summary, a higher rate and broader coverage of reimbursement for TB patients could effectively protect TB patients from catastrophic costs [25].

This study has several limitations. First, the study was restricted to those who seek care in local TB networks; migrant patients may not even seek care due to stigma or financial barriers. Furthermore, we could potentially miss migrant cases who returned to their hometown for diagnosis or treatment even before being notified in Shanghai; and it is difficult to quantify the potential bias associated with these missing cases or its impact on the overall incidence of catastrophic costs among migrants with TB. However, the fact that migrants had better health-care access and resources in Shanghai than in other cities probably minimized the effect of these missing cases. Second, the measures of annual household income and other non-medical expenditures relied on self-reported information, which could be affected by recall biases.

In summary, the high costs of TB care occurred in both internal migrant and resident patients in an urban area with relatively abundant health resources in China. The reimbursement for anti-tuberculous first drugs and diagnostic testings did not significantly reduce the occurrence of catastrophic costs. The low rate of adequate health insurance coverage for migrant TB patients in urban settings highlights a need for available, practical financial assistance for this vulnerable group, as well as crucial health policy to reduce the economic burden for TB patients in China in overall.

## Supplementary information

**Additional file 1 Epidemiology Questionnaire of Tuberculosis Patients Costs Study**

## Data Availability

The data supporting the conclusions of this study are included in the article. The raw datasets generated during and/or analyzed during the current study are available from the corresponding author on reasonable request.

## Abbreviations

TB: Tuberculosis
MDR-TB: Multidrug-resistant tuberculosis
Hukou: Chinese household registration system
CDC: Centers for Disease Control and Prevention
USD: United States Dollar
RMB: Chinese Yuan
WHO: World Health Organiztions
RIF: Rifampin
INH: Isoniazid
IQR: InterQuartile Range
