# Supplementary material for: Catastrophic costs of tuberculosis care in a population with internal migrants in China
Source: BMC Health Serv Res. 2020 Sep 4;20:832. doi: 10.1186/s12913-020-05686-5 (PMC7602335; doi:10.1186/s12913-020-05686-5)
Supplement: Supplementary file 1 — Additional file 1 Epidemiology Questionnaire of Tuberculosis Patients Costs Study [file 12913_2020_5686_MOESM1_ESM.docx]

**Epidemiology Questionnaire of Tuberculosis Patients Costs Study**

Questionnaire Number：________________

Interviewer: ________________

Interviewee__1. Same as patient 2. DOT supporter / guardian 3. other________

Place of interview: ______________ Date: (dd/mm/yy) ___/___/___

**A、Patient information**

1. Patient interview number: ________________

2. Gender: ______（1=male, 2=female）

4. Birth date: _____yy_____mm

5. Resident status: ______ (1=local resident; 2=migrant), if migrant, specify the home province______

6. Education level: ____(1=Primary or less; 2=Junior；3=Senior；4=College；5=University or higher)

7. No. of household members: ________ person(s); Room area_______（m^2^）

8. How much do you estimate was the disposable income of your household per year BEFORE the TB illness? ____________(RMB)

9.What is your occupation before the TB illness？

a. Officials and staff b. General staff/worker c. Technicians

d. Private enterprises e. Student f. Migrant worker

g. Famer h．Other (specify)_______

10. How much do you earn per month BEFORE the TB illness?____________(RMB)

11. Do you have any kind of private or government health/medical insurance scheme today? ______ (1=yes, 2=no)

12. If yes, what type?

a. Medical insurance for urban workers b. Medical insurance for urban residents

c. Commercial health insurance d. Rural cooperative medical insurance

e. Comprehensive insurance of migrant workers f. Others (specify)______________

**B、Treatment history**

13. ____（1=yes，2=no，9=NA）

If yes, what is the initial date your last TB treatment started？ (mm/yy) ___/___

If yes, did you complete your previous TB treatment for at least six months of treatment? ____ (1=yes, 2=no). If No, Why not?

1. Lack of money for treatment costs 2. Drug side effects 3. Moved 4. Distance to health care facility

5. Other (specify): ____________________________________________________

**C、Delay, Pre-diagnostic &Diagnostic Costs**

14. When did you feel sick this time？(dd/mm/yy) ___/___/___

What were the main signs that you were sick? (check all that apply) 1. Fever 2. Weight loss >10% of body weight 3. Night sweats 4. Blood in sputum 5. Cough >15 days 6. Other _________________________

15.When did you start to seek a diagnosis for your illness? (dd/mm/yy)___/___/___

16. When did a health care worker tell you that you have TB (diagnosis date)？ (dd/mm/yy) ___/___/___

17. Health care unit where you were told you have TB _____________________________________________Hospital Other____________________

18. When did you start this anti-TB treatment？(dd/mm/yy) ___/___/___

19. Expected treatment duration ____ 1. (6 months) 2. (8 months) 3. Other_____

| **BEFORE DIAGNOSIS** | **Provider** | **Administrative Costs** (Consultative, registration) | **Tests costs**  (all kind total) | **Drug costs**  (all kind total) | **Travel costs** (return total) | **Food costs** | **Accommodation costs (Total)** | **Sub-Total costs per visit** | **Insurance Reimbursement** If yes: amount, if no n/a |
| --- | --- | --- | --- | --- | --- | --- | --- | --- | --- |
| Visit 1 |  |  |  |  |  |  |  |  |  |
| Visit 2 |  |  |  |  |  |  |  |  |  |
| Visit 3 |  |  |  |  |  |  |  |  |  |
| Visit 4 |  |  |  |  |  |  |  |  |  |
| Visit 5 |  |  |  |  |  |  |  |  |  |
| Visit 6 |  |  |  |  |  |  |  |  |  |
| Visit 7 |  |  |  |  |  |  |  |  |  |
| Visit 8 |  |  |  |  |  |  |  |  |  |
| Total | **N/A** |  |  |  |  |  |  |  |  |
| Did you stop working to seek a disease diagnosis？ If yes，How long_______（months）_______（days） | | | | | | | | | |

20. **About how much did you spend for each of these visits to a health care provider such as a clinic or hospital BEFORE you were diagnosed with TB, including the visit when you actually received your diagnosis?** *For all that don’t apply, mark N/A; Fill one line per visit*

21. **About how much did you spend for each of these visits AFTER you were diagnosed with TB and during the treatment period?** *For all that don’t apply, mark N/A; Fill one line per visit*

| **AFTER DIAGNOSIS** | **Provider** | **Administrative Costs** (Consultative, registration) | **Tests costs**  (all kind total) | **Drug costs**  (all kind total) | **Travel costs** (return total) | **Food costs** | **Accommodation costs (Total)** | **Sub-Total costs per visit** | **Insurance Reimbursement** If yes: amount, if no n/a |
| --- | --- | --- | --- | --- | --- | --- | --- | --- | --- |
| Visit 1 |  |  |  |  |  |  |  |  |  |
| Visit 2 |  |  |  |  |  |  |  |  |  |
| Visit 3 |  |  |  |  |  |  |  |  |  |
| Visit 4 |  |  |  |  |  |  |  |  |  |
| Visit 5 |  |  |  |  |  |  |  |  |  |
| Visit 6 |  |  |  |  |  |  |  |  |  |
| Visit 7 |  |  |  |  |  |  |  |  |  |
| Visit 8 |  |  |  |  |  |  |  |  |  |
| Total | **N/A** |  |  |  |  |  |  |  |  |
| Did you stop working due to TB disease and anti-TB treatment？ If yes，how long _______（months）_______（days） | | | | | | | | | |

Guardian Costs

During the diagnosis and treatment: Circle the correct response or fill in the requested information

| 22. a) Does any family/friend/DOT supporter accompany you on any visits or go in your place to collect your TB drugs? If No, go to 23. | 1. Yes 2. No |
| --- | --- |
| b) If YES, on how many visits has your family/friend/DOT supporter accompanied you or gone in your place? Record pre-diagnosis/diagnosis visits and treatment visits separately  Transport _____ Food ______ Accommodation __________ | _____ Diag. times  ______Treatment times  Total Diag: ____  Total treatment:  ________ |
| Pre-diagnosis/diagnosis costs per visit: | Transport ______  Food ______ Accommodation _____ |
| Costs during treatment per visit: | Transport ______  Food ______ Accommodation _____ |
| c) How much does your friend/family/DOT supporter earn per day? | 1. ___________  2. Doesn’t earn |
| d) Why did someone accompany you? (circle all that apply) | 1. Distance  2. Security  3. Administrative barriers  4. Too ill to travel alone  5. Was required for treatment  6. Other (specify) ____________________ |
|  | |

Hospitalization （Fill the below questionnaire when collecting the final medical invoice）Circle the appropriate answer

| 23. Have you been hospitalized before or during your TB treatment?  If No, go to question 29. | 1. Yes  2. No |
| --- | --- |
| 24. If YES: how many days in total did you stay at the hospital? | __________days |
| 25. Did any family/friend stay with you while in hospital? | 1. Yes  2. No |
| 26. If YES: How many days did he/she stay with you? | __________days  Total: |
| 27. Were there any extra costs for your relative/friend for staying with you at the hospital? | 1. Yes 2. No |
| 28. If Yes, summarize the costs | Transport______  Food__________  Accommodation_______  Other (Describe) ______ |
| 28. How much does your relative/friend who stayed with you, normally earn per day? | 1. ________________  2. Doesn’t earn |

Other Costs

| 29. a) Do you buy any supplements for your diet because of the TB illness (e.g. vitamins; Chinese traditional medicines)? | 1. Yes  2. No |
| --- | --- |
| If YES: How much did you spend on these items approximately? | ______________ |
| 30. a) Do you have any chronic illness for which you are receiving treatment? | 1. Yes  2. No |
| b) If yes: which? List up to three | ______________ |
| c) Are there any additional costs for you because of this other illness besides the costs that you have already mentioned? | 1. Yes  2. No |
| d) If YES: How much are these additional costs on average per month? | 1. Tests: 2. Drugs: 3. Transport: 4. Food: 5. Other:   Total |
